# Eating Macro-Algae (Seaweed): Understanding Factors Driving New Zealand Consumers’ Willingness to Eat and Their Perceived Trust towards Country of Origin

**DOI:** 10.3390/foods13091300

**Published:** 2024-04-24

**Authors:** Meike Rombach, David L. Dean

**Affiliations:** 1 Department of Land Management and Systems, Lincoln University, Lincoln 7647, New Zealand; 2 Center of Excellence-Transformative Agribusiness, Lincoln University, Lincoln 7647, New Zealand; 3 Department of Agribusiness and Markets, Lincoln University, Lincoln 7647, New Zealand; david.dean@lincoln.ac.nz

**Keywords:** consumers, country of origin, seaweed, willingness to eat, PLS-SEM

## Abstract

Macro-algae is an umbrella term for seaweed, which is an important ingredient in many novel food products in New Zealand and other Australasian countries. While attitudes, consumption motivation, knowledge, and socio-demographic consumer profiles have been investigated in specific countries in the region, consumer behavior such as willingness to eat and factors driving this behavior have not yet been explored. Therefore, the present study fills this research gap in a New Zealand context and explores predictors of New Zealand consumers’ willingness to eat macro-algae and their perceived trust towards the countries of origin of these products. The symbolic value of food, health importance, food safety concerns, and food fussiness were the factors under investigation. The work builds on an online questionnaire and a sample of 437 consumers mirroring the New Zealand population in terms of gender, age, and annual household income. Data were collected through an opt-in panel provider in November 2023. The data analysis consisted of descriptive statistics and partial least square structural equation modeling. Results show that health importance and food fussiness tendencies are the strongest predictors of willingness to eat and trustworthiness of the two countries of origin. Best practice recommendations for marketing managers in New Zealand food retail are provided.

## 1. Introduction

In the past decade, plant-based protein sources such as seaweed, otherwise known as macro-algae, have generated consumer interest in Australasian countries such as New Zealand [1,2,3]. Climate change and its negative environmental externalities, food insecurity, and cultural influences have influenced interest in buying and consuming macro-algae [4,5,6,7]. In New Zealand’s culture, people have a strong connection to land and ocean, and food culture is impacted by other island nations across the Pacific, such as Samoa, Fiji, and Tonga, where the consumption of macro-algae is part of their traditional diet [8,9].

Chlorophytes, Phaeophytes, and Rhodophytes are the most common macro-algae types, which are known to be nutritious, rich in fiber, and low in fat [10,11]. In addition to fresh and processed food products containing these species, macro-algae are also an important ingredient in cosmetic products [2,3,12,13]. The macro-algae ingredients for cosmetic and food products available in New Zealand largely come from countries where seaweed aquaculture is widely established, such as Indonesia, China, India, Korea, and the Philippines [13]. New Zealand’s commercial macro-algae production is still in its infancy and rather small in scale [14]. At present, there is pre-production research, and the industry is developing. Trials to date have focussed on determining suitable conditions and practices to produce various macro-algae species [14].

The recent body of literature on macro-algae production and consumption is not extensive. Aspects of consumer behavior are widely unexplored despite the cultural significance of the product in New Zealand and in other Australasian countries. So far, the extant literature has covered Australian consumer markets and discusses socio-demographic profiles of Australian consumers [2,13], as well as attitudes and consumption motivation of younger generational cohorts towards macro-algae [3]. Studies from Samoa are dedicated to consumer motivation and knowledge about macro-algae [8,9]. Tongan and Fijian studies focus mostly on industry development, supply chains, and indigenous knowledge [15,16,17]. Consumer behavior, such as willingness to eat macro-algae, including factors driving this behavior, has yet to be explored in depth. 

Therefore, the present study aims to fill this research gap by investigating (1) predictors driving New Zealand consumers’ willingness to eat macro-algae and (2) their perceived trust towards their country of origin. The outlined literature gap, the emerging character of New Zealand’s macro-algae production, and the importance of export products in consumer markets underpin the importance of this study. In addition, a New Zealand consumer perspective is of value, as consumption patterns are comparable to Australia’s, food chains are similarly structured, and food products available in both countries are provided by similar food retailers [18,19]. New Zealand also shares much with Pacific Island nations as appropriate as almost 382,000 people of New Zealand’s population are Pacifica [20].

Food safety concerns, food fuzziness tendencies, and the importance dedicated to the symbolic value of food and health importance were chosen as relevant predictors. Food safety was chosen to verify findings from Australian studies, which are a common consumer concern in many Western societies [13]. The aspect of health was addressed in all Australasian studies and was therefore included as a predictor to be verified in a New Zealand context [2,3,8,9,13]. The cultural importance and symbolic value of macro-algae have been addressed in in-depth interviews with Samoan and Kiribati consumers [8,9]. Australian research mentioned symbolic value in a food trend context [2,13]. Therefore, in the Australasian context, this is a somewhat new predictor to be explored. Food fussiness has not yet been addressed in an Australasian country context, although two Australian investigations have investigated food neophobia [3,13], the rejection of food, and eating novel food products. To add value to the existing body of literature- the similar but unexplored concept of food fussiness was chosen. Food-fussy consumers may reject novel and familiar food items alike [21]. Therefore, the predictor was included in the study.

**Figure 1:**
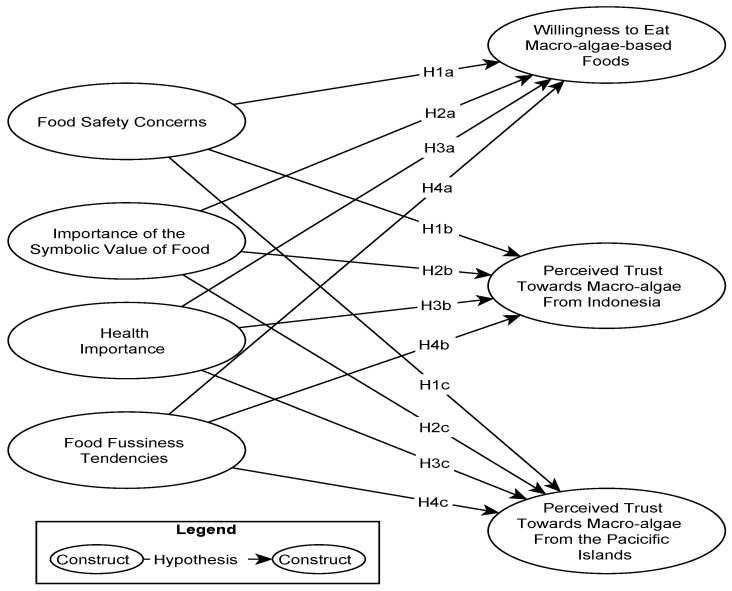
Proposed conceptual model.

In the following sections of this article, a literature review facilitating hypothesis development and a respective conceptual model is presented (see Figure 1). The next section presents the data collection, the survey instrument, and dissemination via an opt-in panel provider, as well as the data analysis, which builds on descriptive statistics and the partial least square structural equation modeling. The final section presents the results and covers the results, discussion, and conclusion. The latter includes recommendations for marketing managers and macro-algae producers, limitations, and s for future works.

## 2. Literature Review and Hypothesis Development

The present research follows an explorative quantitative study by Birch et al. (2019), who investigated drivers and barriers to macro-algae consumption among Australian consumers and developed a framework consisting of predictors. Namely, health importance, responsibility and food safety, food neophobia, symbolic value, and snacking behavior [13]. In this Australian study, all predictors apart from snacking behavior were found to be significant predictors for purchase intentions and consumption frequency [13]. Given that snacking behavior was not found to be a significant predictor, it was not considered in the literature review for the present study. Further changes concerned food neophobia towards food fuzziness, as this change includes the rejection of familiar and novel foods [21]. This change was deemed appropriate as macro-algae may be novel products for a majority but not for all New Zealand consumers. The inclusion of health and symbolic values is underpinned by explorative qualitative studies executed in the Pacific Islands [8,9], where in-depth interviews emphasized the importance of these predictors. Further, a focus on the trustworthiness of countries of origin has been added to the study context, as this concept is not yet widely explored in seaweed consumer studies. It may be influenced by consumer concerns and can influence consumers’ perceptions of product quality [22,23]. Positive or negative emotions and beliefs are associated with a specific country of origin, where a food product is produced, processed, and sold. Based on a product and country-specific image, consumers may evaluate products favorably or unfavorably [22]. Country of origin food studies often explore quality perceptions alongside perceived consumer risk or trust towards buying or trying novel food products [24]. In a macro-algae context, food safety risks related to specific countries of origin appear to be a consumer concern [25].

### 2.1. Food Safety Concerns 

In New Zealand, food safety is a basic consumer right, which is of great importance to New Zealand food retailers and producers [26]. The Ministry of Primary Industries administers the legislation that governs food safety matters in export and domestic markets [26]. In a macro-algae context, Birch et al. (2019) identified food safety concerns as a strong barrier to consumer acceptability and macro-algae consumption [2]. Consumers worry about being negatively affected by pathogens associated with macro-algae, chemical hazards (e.g., iodine, heavy metals), other residues, and allergens that macro-algae can absorb from their environment [27,28,29]. Farming and supply chain studies provide anecdotal evidence that these worries are not unfounded. A Samoan study dedicated to the establishment of aquaculture activities indicates a lack of regulation concerning harvest and environmental protection for areas where macro-algae are growing [30]. Similarly, an Indonesian study focused on macro-algae supply chain management emphasizes that not all farmers follow good practices and that early harvested seaweeds are occasionally contaminated [31]. Therefore, consumers desire certification and verification, which assure them of a hassle-free product that is safe to eat [2,3,32,33]. Consumers with little experience with macro-algae indicate interest in information [2,13]. In a food safety context, research has recognized the impact of the country-of-origin effect [34,35,36]. This effect refers to the positive and negative influence of where a food product is produced and processed on consumers’ attitudes, perceptions, and purchasing decisions [37,38]. Consumers tend to evaluate their country of origin or countries with specific reputations more favorably than other countries [37]. Food scandals and recalls negatively impact trust and lead to stronger bias [35]. Amidst this background, the following hypotheses are proposed:

**Hypothesis 1a (H1a).**  *Food safety concerns negatively impact New Zealand consumers’ willingness to eat macro-algae-based food.*

**Hypothesis 1b (H1b).**  *Food safety concerns negatively impact New Zealand consumers’ perceived trust towards macro-algae from Indonesia.*

**Hypothesis 1c (H1c).**  *Food safety concerns negatively impact New Zealand consumers’ perceived trust towards macro-algae from the Pacific Islands.*

### 2.2. Importance of the Symbolic Value of Food 

Food provides functional and symbolic value to consumers. Their value is associated with the product and impacts their purchase and eating behavior [39]. For symbolic value, the extant literature provides insight into demographic profiles and food consumption and emphasizes cultural and emotional functions associated with eating or specific food products such as macro-algae. Symbolic value is often associated with food memories and personality [40]. Other common consumer associations are nutrition and health importance, culture, lifestyle, and environment [41,42,43]. In New Zealand, macro-algae have symbolic value and cultural importance to the Māori (indigenous population), as the maco-algae harvest provided a source of income to the indigenous population during the Second World War [44,45]. An Australian study reports that females and health-conscious consumers with higher household incomes and educational backgrounds tend to assign symbolic value to food and are more likely to consume seaweed [2]. Given that the country-of-origin effects impact perception and hence value associated with the macro-algae product, the following hypotheses are proposed.

**Hypothesis 2a (H2a).**  *The importance of the symbolic value of food positively impacts New Zealand consumers’ willingness to eat macro-algae-based food.*

**Hypothesis 2b (H2b).**  *The importance of the symbolic value of food positively impacts New Zealand consumers’ perceived trust towards macro-algae from Indonesia.*

**Hypothesis 2c (H2c).**  *The importance of the symbolic value of food positively impacts New Zealand consumers’ perceived trust towards macro-algae from the Pacific Islands.*

### 2.3. Health Importance

Macro algae are so-called superfoods that are high in fiber and rich in micronutrients and antioxidants [46]. The product appears to be beneficial for health improvements related to weight loss, high blood pressure, diabetes, and bone and digestive health [2]. Consumers in New Zealand and other Australasian countries are becoming increasingly more health conscious. Birch (2019) and Young (2022) found that in Australia, in particular, people with higher income and higher education are health conscious and willing to eat macro-algae [2,3]. Findings from Samoa and Kiribati indicate that consumers who are health and budget-conscious are appreciative of macro-algae consumption [8]. Samoan consumers favor the species Caulerpa and Halymenia due to their taste and nutritional properties [9]. Samoan consumers in the age range of 45 years and older appeared to be more health conscious than others and willing to consume macro-algae, as these consumers have more traditional knowledge related to plants and health [9]. Amidst this background, it is assumed health and country of origin beliefs impact consumer behavior and their trust. It is hypothesized that:

**Hypothesis 3a (H3a).**  *Health importance positively impacts New Zealand consumers’ willingness to eat macro-algae-based food.*

**Hypothesis 3b (H3b).**  *Health importance positively impacts New Zealand consumers’ perceived trust towards macro-algae from Indonesia.*

**Hypothesis 3c (H3c).**  *Health importance positively impacts New Zealand consumers’ perceived trust towards macro-algae from the Pacific Islands.*

### 2.4. Food Fussiness Tendencies

Food fussiness refers to the aversion and refusal to eat foods that are often widely accepted [47]. Food fussiness is also known as pickiness and can be present in children and adults [48,49]. Consumers who are food fussy tend to reject foods based on sensory characteristics inherent to the product [43]. For macro-algae, this may include taste, color, and texture, as the specific species and their processing impact sensory characteristics [50]. For instance, cooking can lead to changes in color and texture [50]. In addition, food-fussy consumers are often less likely to enjoy eating healthy food items [43]. Since the country-of-origin effect may impact the consumer perception of macro-algae and could intensify the food fussiness tendencies, the following hypotheses are proposed:

**Hypothesis 4a (H4a).**  *Food fussiness tendencies impact New Zealand consumers’ willingness to eat macro-algae-based food.*

**Hypothesis 4b (H4b).**  *Food fussiness tendencies impact New Zealand consumers’ perceived trust towards macro-algae from Indonesia.*

**Hypothesis 4c (H4c).**  *Food fussiness tendencies impact New Zealand consumers’ perceived trust towards macro-algae from the Pacific Islands.*

## 3. Material and Methods

### 3.1. Data Collection and Sampling Methods

Data for this research were gathered through an online survey in November 2023. Utilizing the digital Qualtrics XM platform, the survey aimed to explore consumer behaviors concerning macro-algae [51]. The study examined variables including the symbolic value of food, health importance, food safety concerns, and food fussiness. The questionnaire was tailored to the unique context of macro-algae, building on existing research [2,3,13,52,53]. Items were measured on a 7-point Likert scale measuring agreement. The health importance items were derived from Gould’s (1990) health-consciousness scale [54] and recent studies on superfoods by Wiedenroth and Otter (2021), a product widely bought for their health benefits [52,53]. Food safety concerns and symbolic value were adapted from Birch et al. (2019) [2], whereas food fuzziness items were built on the work of Harris (2019) [55]. Willingness to eat was developed by the authors to measure the construct across a variety of macro-algae-based foods, capturing strength and breadth of willingness. ‘Trust towards Indonesia and the Pacific Islands as countries of origin for macro algae-based products was created by the authors and was measured with 0–100 sliding scales ranging from ‘Not at all trustworthy’ (0) to ‘Extremely trustworthy’ (100). Indonesia and the Pacific Islands were chosen as New Zealand consumers are likely to be familiar with these countries of origin. In New Zealand, approximately 9% of the population identifies as Pacifica [20]. In addition, the Pacific Islands are important trade partners to New Zealand [56]. Indonesia is a major macro-algae producer supplying macro-algae-based food and cosmetic products to the New Zealand market [13], with an active trade relationship with New Zealand when it comes to food and beverages [57]. As products are widely available in NZ consumer markets, Indonesia was chosen over other leading producers, such as China and Korea.

To obtain an appropriate sample, particular selection parameters were set. The study required participants to be 18 years or older, New Zealand residents, and responsible for their household’s food shopping. In addition, we only targeted consumers with some interest and experience in macro-algae. Survey participants who had no interest in buying or eating macro-algae-based products were excluded from the survey. The survey was disseminated through Qualtrics as an opt-in panel provider that recruited the study participants [58]. Initially, 450 survey responses were obtained. Considering the average completion duration of 20 min, a total of 13 submissions were deemed unfit for the analysis. The reasons for omitting responses included incomplete answers or indications of rushed responses [59]. The socio-demographic profiles of the 437 survey participants are displayed in Table 1. 

The sample mirrors the New Zealand population relatively accurately in terms of gender, age, and income (see Table 1) [60]. Only the low and high-income categories had major differences from the latest NZ Census. In terms of their dietary requirements, 80.6% of the respondents indicated being omnivores, 7.6% acknowledged being impacted by allergies and intolerances and thus avoided seafood, gluten, fructose, or lactose, and 2.4% of the respondents stated that their diet was kosher, halal, or otherwise following their religious beliefs. Furthermore, 5.7% of the survey followed a semi-vegetarian diet and occasionally ate fish or chicken, and 3.7% followed a vegetarian or vegan lifestyle and avoided eating meat and animal-based products. 

Following Hair et al. (2022), the sample size of 437 responses is appropriate for the Partial Least Squares Structural Equation Modeling (PLS-SEM) analysis [61]. It is recommended that the ’10-times rule’ be used to determine a suitable sample size for a PLS-SEM study. According to this rule, the sample should be at least ten times greater than the maximum number of structural paths directed at any latent variable in the model [61]. Consequently, the minimum sample size required for the research was established at 40. Therefore, the final sample size used in the analysis was deemed sufficiently large for the analysis.

### 3.2. Data Analysis

The data analysis for this study was conducted in a systematic three-stage process. In the first stage, SPSS 28 software was utilized to conduct descriptive statistical analyses to characterize the sample. The second and third stages involved the use of SmartPLS 4, a tool specifically designed for PLS-SEM [59]. PLS-SEM is a statistical technique used for complex multivariate analyses, particularly useful in exploring relationships between observed and latent variables [61,62].

The second stage involved assessing the reliability of the measurement model, following established criteria. In survey research, a series of questions (items) that measure several aspects of a concept (construct) can be combined into a multi-item scale to measure the construct. These scale items need to be sufficiently related to each other (reliability); they need to measure the same construct (convergent validity), and different scales should not measure the same construct (discriminant validity). Reliability, a measure of consistency, was determined using Cronbach’s alpha and composite reliability scores. Scores exceeding 0.6 in both these metrics were deemed satisfactory, aligning with the standards set in previous research [50]. Convergent validity, which assesses whether different measures of the same concept are correlated, was confirmed when item/scale factor loadings exceeded 0.4 and the average variance extracted (AVE) values were above 0.6 [59,60,61]. 

Discriminant validity is confirmed using the Heterotrait–Monotrait ratio (HTMT) and the Fornell–Larcker criteria, with the former requiring values below 0.9 [61,63]. Additionally, to ensure the reliability of these findings, multicollinearity was checked using Variance Inflation Factor (VIF) scores, with the accepted threshold being below 5, as higher values could indicate problems in the analysis [61,62,63,64].

The third stage of analysis involved the structural model, which tests the hypothesized relationships between constructs [61]. This was done through bootstrapping with 10,000 iterations, a method used to estimate the distribution of a statistic by resampling with replacement from the data. This approach helps in assessing the significance of path relationships [61]. The model’s performance was then evaluated using several metrics, including the goodness of fit (GoF), normed fit index (NFI), and standardized root mean square residual (SRMR). GoF and NFI scores provide an overall indication of the model’s fit to the data, while SRMR values, which should ideally be at or below 0.08, with a maximum threshold of 0.1, offer insights into the model’s residuals [61].

Finally, the model’s explanatory power (R^2^) and predictive relevance (Q^2^) were benchmarked [61]. The R^2^ values, indicative of the model’s ability to explain the variance in the dependent variables, were categorized into different magnitudes of effect: 0.25 for small, 0.5 for moderate, and 0.75 for large effects. Similarly, Q^2^ values, which reflect the model’s predictive accuracy, were evaluated, with values above 0 indicating acceptable predictive relevance, above 0.25 indicating moderate relevance, and values above 0.5 signifying high levels of predictive relevance [61]. In conclusion, the third stage confirms whether the overall model is fit for purpose (by evaluating GoF, NFI, & SRMR), it tests the model’s ability to explain and predict the dependent variables (by evaluating R^2^ and Q^2^), and it tests whether the proposed relationships are statistically significant.

## 4. Results and Discussion

Table 2 indicates that Cronbach’s Alpha and Composite Reliability are in line with Hair et al. (2022) and their recommendations. All values are well above the threshold of 0.6 [61,62]. These findings indicate strong reliability for the constructs under investigation. Moreover, factor loadings were above the 0.6 threshold, and the Average Variance Extracted (AVE) exceeded the threshold value of 0.5 [61], indicating that the model is internally consistent and fulfilling the conditions for convergent validity [61]. Descriptive statistics for single-item measures are presented in Table 3.

Table 4 displays the Fornell–Larcker criterion and the HTMT ratios, as important criteria for discriminant validity. These criteria for discriminant validity were satisfied, with cross-loadings less than the diagonal values in the Fornell–Larcker criterion, and the HTMT ratios below their threshold value of 0.9. The average VIF value was 1.200 and was therefore below the recommended threshold of 5. This indicates that the model is not affected by multicollinearity problems [61].

The results for the structural model show a GoF of 0.311, an NFI of 0.778, and an SRMR of 0.067, indicating adequate model fit. For its explanatory power, the model shows an R^2^ value of 0.153, explaining 15.3% of the variance in consumers’ willingness to eat macro-algae-based foods, and an R^2^ of 0.104 and 0.067, accounting for 10.4% and 6.7% of the perceived trust towards Indonesia and the Pacific Islands as country of origin of macro-algae-based foods. The model’s predictive relevance was confirmed, with all three Q^2^ values being above zero, and an average Q^2^ value of 0.104 indicates the model’s acceptable but somewhat weak predictive relevance and explanatory power as indicated by the R^2^ values. The results from hypothesis testing are displayed in Table 5, showing support for most of the hypotheses. 

The study found a significant, positive relationship between food safety concerns and consumers’ willingness to eat macro-algae-based. As indicated in Hypotheses H1a, a negative relationship was anticipated. These findings diverge from the recent body of literature, which indicates that consumers are concerned about bacterial and chemical contamination as a risk to food safety [2]. New Zealand consumers appear not to associate food safety risks with macro-algae. In addition, food safety standards in NZ are relatively high. Interestingly, the relationships between food safety concerns and perceived trust towards Indonesia and the Pacific Islands as countries of origin were not found to be significant (see hypotheses H1b and H1c). Similarly, the importance of the symbolic value of food resulted in non-significant relationships between willingness to eat macro-algae-based food as well as perceived trust towards the macro-algae products from Indonesia and the Pacific Islands, indicating no support for Hypotheses H2a–H2c. The non-significant relationships may be explained by the fact that macro-algae are not yet common in everyday diets in NZ. Hence, a majority of consumers may be less able to identify and attribute value and meaning to the product. However, significant positive relationships were found for health importance and willingness to eat macro-algae-based food, as well as perceived trust towards Indonesia and the Pacific Islands as countries of origin for macro-algae-based products, showing support for hypotheses H3a–H3c. These findings confirm previous studies. Consumer studies dedicated to macro-algae consumption found that consumers who have higher education or high income were more likely to report health importance as a factor influencing macro-algae purchase and consumption [2]. Flavor, nutrient composition, and health benefits have been reported as the main drivers for consumers influencing macro-algae consumption [2,3,8,9]. In the macro-algae context, the relationship between health importance and preferences and trust toward country of origin is not yet widely explored. However, marketing studies dedicated to food packaging show that the importance of diet for health reasons also increases the importance of the country of origin for consumers [65]. Significant negative relationships were found for food fussiness tendencies and willingness to eat macro-algae-based food, as well as perceived trust in Indonesia and the Pacific Islands as countries of origin of macro-algae-based products, supporting hypotheses H4a–H4c. Fussy eaters are known to be more monotonous and rigid about the foods they consume [43]. Foods should appear and be prepared so they are aligned with their preferences and habits; otherwise, they may be rejected [47]. Food fussiness is a form of selective eating where people even refuse familiar food products that do not match their expectations of presentation, as well as new food products that they are unfamiliar with. Given that many NZ consumers may consider macro-algae a rather new food product, the potential unfamiliarity with the product could explain the negative relationships. Further research could explore the role of familiarity. 

## 5. Conclusions

The results presented in this study are of importance to marketers in New Zealand’s food industry. Given that health importance and food fussiness were the strongest predictors for willingness to eat macro-algae and perceived trust toward macro-algae from both the Pacific islands and Indonesia, retailers and marketers may consider the impact of these attitudinal constructs. For New Zealand retailers targeting both fussy and regular sea-vegetable consumers, marketers should emphasize the nutritional and health benefits of the product. In addition, products should provide QR codes and labeling from verification from certification bodies so they can be assured that production and processing follow the highest health safety standards. For fussy eaters, producing macro-algae products that are more familiar to consumers may make the product more appealing. For snacks such as crackers and candy, not displaying the natural appearance of macro-algae may be a good strategy. Avoiding attributes that fussy consumers may reject may increase opportunities to gain product acceptance. Regarding countries of origin, New Zealand consumers seem to trust both Indonesia and the Pacific Islands as macro-algae producers. And this trust is strengthened for health-conscious consumers and weakened for fussy eaters. This finding suggests that the country of origin may offer a suitable point of differentiation for macro-algae marketers.

Besides these practical implications, the current manuscript adds to the recent body of literature by filling a literature gap that is, at present, widely unexplored. Future work could address different ethnicities living in NZ and compare consumption habits, preferences, and willingness to pay across different Asian and Pacifica groups living in New Zealand. Also, cross-country comparisons with other Oceanian nations could be a fruitful direction. A best-worst scaling investigation and latent class analysis may help to understand product attribute preferences of macro-algae and allow the development of consumer profiles. Food disgust and food safety are promising avenues for future research. Food safety should be investigated in the context of risk information and focus on food safety risks specifically associated with macro-algae. This would fill a literature gap as recent quantitative investigations focus on the context of food safety more in general. Qualitative and mixed method work could focus on social media reporting and investigate online word of mouth and loyalty towards specific macro-algae products. Lastly, it should be acknowledged that the sensory evaluation of macro-algae-based food and beverages is an important determinant of consumers’ willingness to consume again. This research did not expect that consumers had consumption experience with all of the macro-algae-based products examined, so sensory evaluation was not considered. However, integrating sensory evaluations in future research could provide valuable results. A combination of product tasting and eye-tracking may be useful to explore taste, taste, texture, scent, appearance, and commercial product attributes, which are likely to influence consumer preferences.

## Figures and Tables

**Table 1:** Sample demographics.

	Freq	%	NZ Census
Age			
18–24	62	14.2	12.2
25–34	79	18.1	18.4
35–44	72	16.5	16.3
45–54	77	17.6	17.5
55–64	62	14.2	15.7
65+	85	19.5	19.9
Total	437	100.0	100
Household Annual Income			
$0 to $24,999	50	11.4	19.0
$25,000 to $49,999	125	28.6	40.0
$50,000 to $74,999	116	26.5	24.0
$75,000 to $99.999	65	14.9	11.0
$100,000 or higher	81	18.5	6.0
Total	437	100.0	100
Gender			
Male	213	48.8	49.4
Female	224	51.3	50.7
Total	437	100.0	100

**Table 2:** Scale loadings, reliabilities, and convergent validity.

Scales and Items	Factor Loadings	Cronbach’s Alpha	Composite Reliability	AVE
Food fussiness tendencies		0.906	0.934	0.780
I have been called a picky eater	0.880			
I think that many foods are disgusting	0.876			
I find most foods distasteful	0.856			
I consider myself to be a picky eater	0.918			
Health importance		0.878	0.903	0.539
I am aware of my health as I go through the day	0.684			
I notice how I feel physically as I go through the day	0.689			
I am very involved with my health	0.763			
I am constantly examining my health	0.709			
I am alert to changes in my health	0.788			
I am generally alert to my inner feelings about my health	0.786			
I reflect on my health a lot	0.777			
I am very self-conscious about my health	0.667			
Food safety concerns		0.826	0.875	0.584
I want to know about the conditions surrounding the production of the food I buy	0.746			
I try to choose food that is produced in a sustainable way	0.789			
I’m concerned about the amount of artificial additives and preservatives in food	0.775			
It is important to understand the environmental impact of our eating habits	0.822			
The quality and safety of food nowadays concern me	0.681			
Importance dedicated to the symbolic value of food		0.864	0.917	0.785
What and where someone eats, says something about who they are as a person	0.860			
The food you eat is an expression of your personality	0.901			
You can tell a lot about a person by what they eat	0.897			
Willingness to eat macro-algae-based foods		0.835	0.876	0.503
Crackers or Cookies	0.710			
Soup	0.633			
Salad	0.683			
Smoothy or Shake	0.765			
Jelly or Candy/Lolly	0.715			
Tea	0.743			
Supplements	0.707			

**Table 3:** Descriptive statistics for single-item measures.

Items	Mean	Std. Dev.
Perceived trust towards countries of origin		
Perceived trust towards macro-algae from the Pacific Islands	62.973	26.425
Perceived trust towards macro-algae from Indonesia	81.488	20.122

**Table 4:** Scale Discriminant Validity.

Fornell-Larcker Criterion	A	B	C	D	E	F	G
(A) Food Safety Concerns	0.764						
(B) Importance of Symbolic Value of Food	0.391	0.886					
(C) Health Importance	0.455	0.318	0.734				
(D) Food Fussiness Tendencies	−0.138	0.081	−0.103	0.883			
(E) Willingness to eat macro-algae-based foods	0.289	0.132	0.244	−0.271	0.709		
(F) Perceived trust towards Indonesia	0.165	0.111	0.278	−0.186	0.299	1	
(G) Perceived trust towards the Pacific Islands	0.137	0.017	0.174	−0.201	0.153	0.496	1
**HTMT**	**A**	**B**	**C**	**D**	**E**	**F**	**G**
(A) Food Safety Concerns							
(B) Importance of Symbolic Value of Food	0.457						
(C) Health Importance	0.522	0.372					
(D) Food Fussiness Tendencies	0.159	0.094	0.17				
(E) Willingness to eat macro-algae-based foods	0.329	0.159	0.274	0.303			
(F) Perceived trust towards Indonesia	0.172	0.12	0.289	0.191	0.324		
(G) Perceived trust towards the Pacific Islands	0.140	0.017	0.174	0.204	0.162	0.496	

**Table 5:** Coefficients for Hypothesized Paths.

Hypothesised Relationship	Coefficient	T Stat	*p* Value
H1a: Food safety concerns -> Willingness to eat macro-algae-based food	**0.186**	3.311	0.001
H1b: Food safety concerns -> Perceived trust towards Indonesia	0.018	0.3	0.765
H1c: Food safety concerns -> Perceived trust towards the Pacific Islands	0.064	1.138	0.255
H2a: Importance dedicated to the symbolic value of food -> Willingness to eat macro-algae-based food	0.040	0.659	0.510
H2b: Importance dedicated to the symbolic value of food-> Perceived trust towards Indonesia	0.041	0.796	0.426
H2c: Importance dedicated to the symbolic value of food-> Perceived trust towards the Pacific Islands	−0.038	0.759	0.448
H3a: Health importance -> Willingness to eat macro-algae-based food	**0.122**	2.451	0.014
H3b: Health importance-> Perceived trust towards Indonesia	**0.240**	4.448	<0.001
H3c: Health importance-> Perceived trust towards the Pacific Islands	**0.139**	2.64	0.008
H4a: Food fussiness tendencies -> Willingness to eat macro-algae-based food	**−0.236**	5.096	<0.001
H4b: Food fussiness tendencies -> Perceived trust towards Indonesia	**−0.162**	3.48	0.001
H4c: Food fussiness tendencies -> Perceived trust towards the Pacific Islands	**−0.174**	3.639	<0.001

**Bold** = *p* ≦ 0.05.

## Data Availability

The original contributions presented in the study are included in the article, further inquiries can be directed to the corresponding author.
